# Osthole Antagonizes Microglial Activation in an NRF2-Dependent Manner

**DOI:** 10.3390/molecules28020507

**Published:** 2023-01-04

**Authors:** Chuan-Hsiu Liu, Mei-Ying Chen, Yueh-Hsiung Kuo, Jack Cheng, Li-Zhong Chang, Meng-Shiun Chang, Tsai-Ni Chuang, Wen-Tsong Hsieh, Yan-Ru Xiao, Bor-Tsang Wu, Wei-Yong Lin, Hsin-Ping Liu

**Affiliations:** 1 Graduate Institute of Chinese Medicine, China Medical University, Taichung 40402, Taiwan; 2 School of Chinese Medicine, China Medical University, Taichung 40402, Taiwan; 3 Graduate Institute of Integrated Medicine, College of Chinese Medicine, China Medical University, Taichung 40402, Taiwan; 4 Department of Chinese Pharmaceutical Sciences and Chinese Medicine Resources, College of Chinese Medicine, China Medical University, Taichung 40402, Taiwan; 5 Department of Biotechnology, Asia University, Taichung 41354, Taiwan; 6 Chinese Medicine Research Center, China Medical University, Taichung 40402, Taiwan; 7 Department of Medical Research, China Medical University Hospital, Taichung 40447, Taiwan; 8 Department of Pharmacology, School of Medicine, China Medical University, Taichung 40402, Taiwan; 9 Department of Senior Service Management, National Taichung University of Science and Technology, Taichung City 40343, Taiwan; 10 Graduate Institute of Acupuncture Science, College of Chinese Medicine, China Medical University, Taichung 40402, Taiwan

**Keywords:** osthole, NRF2, microglia

## Abstract

Microglia are neuroglia in the brain with an innate immune function and participate in the progress of neurodegenerative diseases. Osthole (OST) is a coumarin derivative extracted from *Cnidium monnieri* and bears a microglia-antagonizing ability. However, the underlying mechanism of the antagonism is not clear. The lipopolysaccharides-induced microglial BV2 cell line and amyloid-overexpressing fruit fly were used as models to study OST treatment. We found that OST treatment is sufficient to evoke NRF2 cascade under an LPS-induced inflammatory environment, and silencing NRF2 is sufficient to abolish the process. Moreover, we found that OST is sufficient to antagonize microglial activation in both LPS-induced BV2 cells and Aβ-overexpressing fruit flies, and silencing NRF2 abolishes OST’s antagonism. Furthermore, OST treatment rescued survival, climbing, and the learning ability of Aβ-overexpressing fruit flies and relieved oxidative stress. In conclusion, we proved that OST antagonizes microglial activation induced by either LPS or Aβ and that NRF2 is necessary for OST’s antagonism.

## 1. Introduction

Reactive oxygen species (ROS) homeostasis is critical for cell signaling, metabolism, development, and immune function [1]. ROS homeostasis disturbed by the accumulation of aggregated proteins is believed to be involved in the progression of neurodegenerative diseases (NDs), such as amyotrophic lateral sclerosis (ALS), Parkinson’s disease (PD), and Alzheimer’s disease (AD) [2]. These NDs are characterized by the activation of microglia along with neurodegeneration caused by oxidative damage [3]. Microglia are resident macrophage cells in the central nervous system (CNS) and act as an innate immune defense by scavenging pathogens and damaged neurons [4]. Notably, ROS and cytokines released by the microglia might be the cause of neurodegeneration [3]. Therefore, antioxidants are candidates for drug discovery against neurodegeneration development [5], in particular targeting the mechanism of ROS homeostasis [6].

The nuclear factor erythroid 2–related factor 2 (NRF2) is a transcription factor that critically regulates ROS homeostasis. At low levels of ROS, NRF2 tightly binds with the Kelch-like ECH-associated protein 1 (KEAP1) complex and thus briefly goes to ubiquitination and degradation [7]. However, in the case of high levels of ROS, NRF2 disassociates with KEAP1 and translocates into the nucleus, and it acts as a transcription factor by binding to ARE domains and activating the expression of antioxidant proteins, including heme oxygenase-1 (HO-1), NAD(P)H quinone dehydrogenase 1 (NQO1), superoxide dismutase type 1 (SOD1), and catalase (CAT) [8]. Furthermore, due to the pivoting role of NRF2 in ROS homeostasis, compounds activating NRF2 cascades are targets of drug discovery against neurodegeneration [9].

Osthole (OST, 7-methoxy-8-(3-methylbut-2-enyl)chromen-2-one, PubChem ID: 10228), also known as osthol, is a coumarin derivative extracted from several medicinal plants like *Cnidium monnieri*. In Chinese medicine, Cnidium is characterized by its taste of bitterness, its property of warmth, running through the meridian of the kidney, and its ability to destroy parasites and relieve itching, to invigorate the kidney and support yang. However, the toxicity of a high dosage should be cautioned against [10]. In modern clinical research, osthole is applied in several fields, including the protection of the cerebrum from ischemia-reperfusion injury [11], stimulation of osteoblast differentiation and bone formation [12], alleviation of hyperglycemia [13], suppression of the secretion of hepatitis B virus [14], and inhibition of the growth of hepatocellular carcinoma [15].

Previous studies have shown the beneficial activities of OST, including antitumor, anti-inflammation, and neuroprotective ones [16]. Interestingly, OST has been reported to antagonize microglial activation under an inflammatory/oxidative environment [17]; however, of several possible underlying pathway candidates, it is still unclear which one is necessary. These pathway candidates include (1) inhibiting calcium entry and elevating cGMP levels [18], (2) modulating PI3K/Akt/mTOR signaling [17], (3) suppressing Notch signaling [19], (4) down-regulating NF-κB signaling [20], (5) inhibiting NLRP3 inflammasome [21], and (6) activating NRF2 signaling [22]. These pathways cover the regulation of inflammatory response, intrinsic immunity, and ROS scavenging, and they are interrelated. Identifying the key pathway, which is necessary for OST to antagonize microglial activation, will make the role of ROS scavenging clearer and benefit rational drug design using OST derivatives in the future.

To answer the question of whether NRF2 signaling is indispensable for OST to suppress microglial activation under an inflammatory/oxidative environment, we tested whether NRF2-RNAi abolishes this reaction in lipopolysaccharide (LPS)-treated BV-2 microglial cells. We further showed the OST’s efficacy in ameliorating AD symptoms and ROS burden in an Aβ-overexpressing fruit fly model.

## 2. Results

### 2.1. OST Activated the Nrf2/HO-1 Signaling Pathway in LPS-Stimulated BV2 Cells

**Figure 1:**
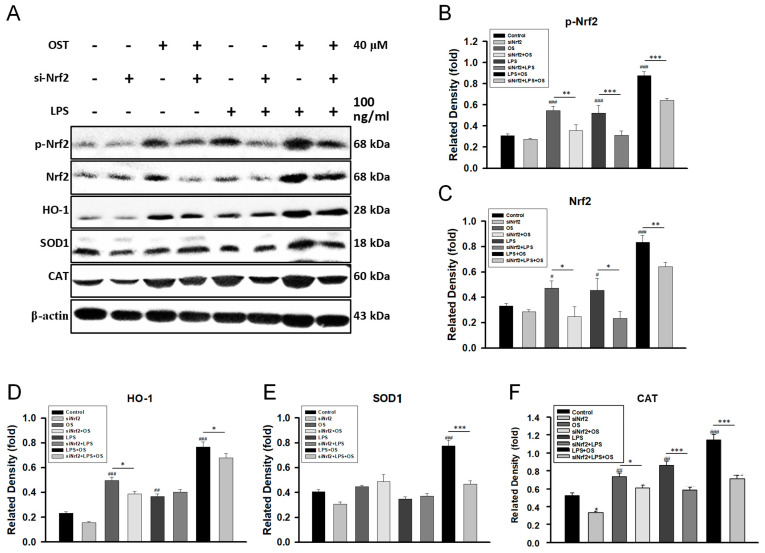
OST activated the Nrf2/HO-1 signaling pathway, but it was reversed by the Nrf2 siRNA transfection in BV2 cells. (**A**) Mock (control) or Nrf2 siRNA were transfected into BV2 cells. Cells were treated with 40 μM OST for 1 h before being stimulated with LPS for 24 h. The protein expressions were analyzed with a western blot assay. The expression of related proteins, including (**B**) p-Nrf2, (**C**) Nrf2, (**D**) HO-1, (**E**) SOD1, and (**F**) CAT were shown individually. All results were expressed as folds mean ± SEM compared with β-actin of three independent experiments (n = 3). # *p* < 0.05, ## *p* < 0.01 ### *p* < 0.001 compared with the control (mock) group; * *p* < 0.05, ** *p* < 0.01, and *** *p* < 0.001 compared with the counterpart of the mock-treated group.

**Figure 2:**
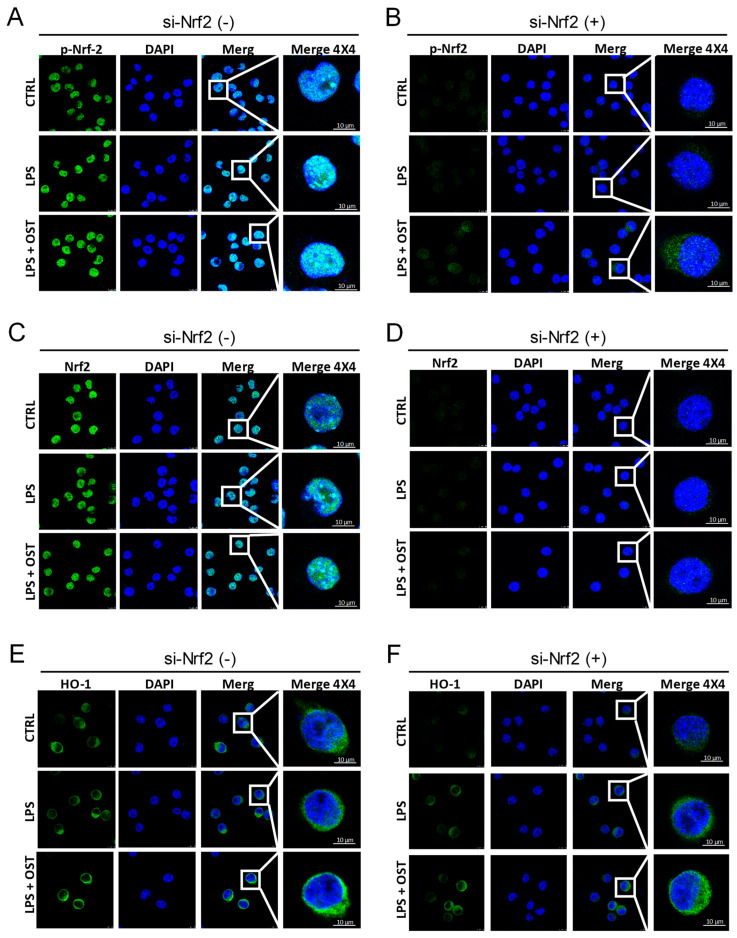
Illustrative images of immunofluorescence staining showed the effect of OST on the expression of p-Nrf2 in LPS-stimulated (**A**) BV2 cells and (**B**) Nrf2 siRNA-treated BV2 cells. The expression of Nrf2 in LPS-stimulated (**C**) BV2 cells and (**D**) Nrf2 siRNA-treated BV2 cells. The expression of HO-1 in LPS-stimulated (**E**) BV2 cells and (**F**) Nrf2 siRNA-treated BV2 cells.

To test whether OST activates the Nrf2/HO-1 signaling pathway in LPS-stimulated BV2 cells, we applied OST to a well-established BV2 cell line with the stimulus of LPS. BV2 is a microglial cell line derived from C57/BL6 mice and serves as a substitute for primary microglia in many experimental settings [23]. LPS are prototypical endotoxins derived from Gram-negative bacteria’s cell walls and serve as a well-established inducer of inflammation [24]. As shown in Figure 1A, the protein expression of the Nrf2/HO-1 signaling pathway, including phosphorylated and total Nrf2, HO-1, SOD1, and CAT, was significantly increased by OST. The quantification is shown in Figure 1B–F. Furthermore, OST increased the nuclear translocation of p-Nrf2 in BV2 cells (Figure 2A,B), but not Nrf2 (Figure 2C,D), and increased the accumulation of HO-1 in the perinuclear space (Figure 2E,F). Thus, OST activated the Nrf2/HO-1 signaling pathway in BV2 cells.

### 2.2. Nrf2 siRNA Suppressed OST-Induced Antagonism toward Microglial Activation

**Figure 3:**
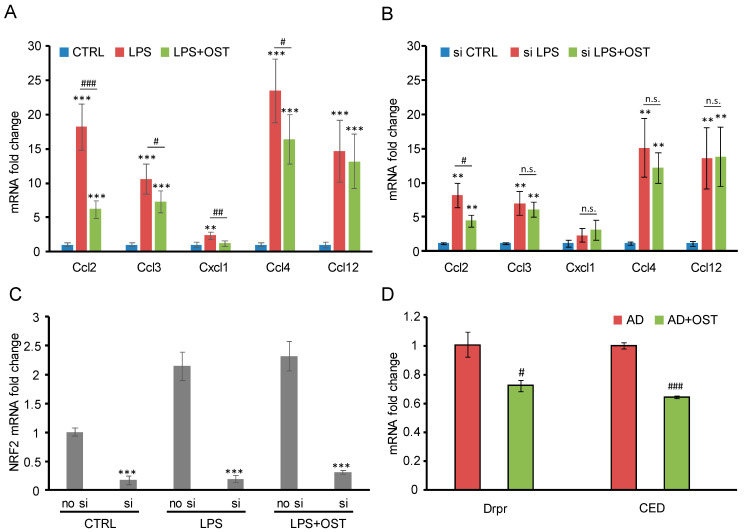
Nrf2 siRNA suppressed OST-induced antagonism toward microglial activation provoked by LPS. (**A**) OST antagonized LPS-induced expression of microglial activation markers in BV2 cells, while (**B**) this phenomenon was suppressed by Nrf2 siRNA. (**C**) Nrf2 siRNA effectively silenced NRF2 expression. (**D**) OST antagonized amyloid-beta (Aβ)-induced expression of microglial activation markers in Alzheimer’s disease model of a fruit fly. ** and *** denote *p* < 0.01 and 0.001 of Student’s *t*-test compared to CTRL group, respectively. #, ##, and ### denote *p* < 0.05, 0.01, and 0.001 of Student’s *t*-test compared to LPS-treated BV2 cells in (**A**), (**B**), or to AD flies in (**D**), respectively. The error bar stands for the standard error of the mean. n.s. stands for non-significant.

Microglial activation is an innate immune response in the central nervous system, scavenging pathogens [25] or facilitating neurogenesis [26]. However, microglial activation itself is associated with neurodegenerative diseases as a pathogenic driver, which destroys neuronal circuits. Therefore, clarifying the mechanism that antagonizes microglial activation is a strategy of drug development against neurodegenerative diseases [27]. Previous studies have shown that OST antagonizes microglial activation; however, there are several underlying candidate mechanisms, as described in the introduction. To test whether OST antagonizes microglial activation through the Nrf2/HO-1 signaling pathway, microglial activation markers, such as the gene expression of Ccl2, Ccl3, Cxcl1, Ccl4, and Ccl12, were measured in LPS-stimulated BV2 cells. As shown in Figure 3A, LPS induced the gene expression of Ccl2, Ccl3, Cxcl1, Ccl4, and Ccl12, while OST treatment significantly decreased the gain of Ccl2, Ccl3, Cxcl1, and Ccl4. Moreover, by treating Nrf2 siRNA, the antagonizing ability of OST against microglial activation was largely abolished (Figure 3B). The effectiveness of Nrf2 siRNA in silencing the expression of Nrf2 was confirmed (Figure 3C).

### 2.3. OST Ameliorated Alzheimer’s Disease Symptoms in Drosophila Animal Model

**Figure 4:**
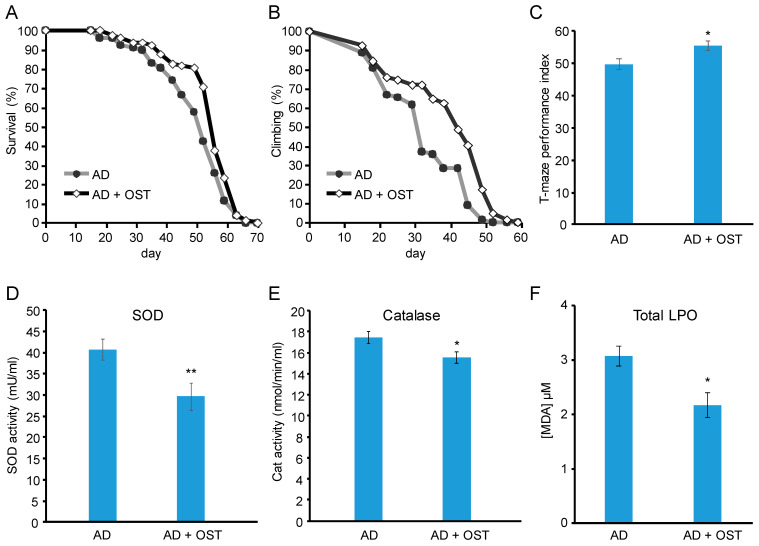
OST ameliorated Alzheimer’s disease (AD) symptoms and lowered oxidative stress in the *Drosophila* animal model. (**A**) Survival, (**B**) climbing ability, (**C**) learning ability, (**D**) superoxide dismutase (SOD) activity, (**E**) catalase activity, and (**F**) total lipid peroxidation (LPO). In the figures, “AD” stands for the AD *Drosophila* model with a pan-neuronal expression of amyloid beta (Aβ), while “AD + OST” stands for the OST-treated AD *Drosophila* model. * and ** denote *p* < 0.05 and 0.01 of Student’s *t*-test, respectively. The error bar stands for the standard error of the mean.

To test whether OST antagonizes microglial activation induced by other pathogens besides LPS in other organisms besides mice, we utilized the Alzheimer’s disease (AD) *Drosophila* model, whose neural system harbors the over-expression of human amyloid beta (Aβ), with symptoms of shortened survival, diminished climbing ability, defects of learning ability, and a higher oxidative burden [28]. By treating the AD Drosophila model with OST, we found that markers of microglial activation, such as the gene expression of Drpr and Ced [29], decreased (Figure 3D) and that AD symptoms, including survival (Figure 4A), climbing ability (Figure 4B), and learning ability (Figure 4C), were ameliorated. Moreover, the oxidative burden was decreased (Figure 4D–F).

## 3. Discussion

This study found that OST treatment is sufficient to evoke an NRF2 cascade under an LPS-induced inflammatory environment and that silencing NRF2 is sufficient to abolish the process. This means that the anti-inflammatory activity of OST is largely dependent on the NRF2 cascade. Moreover, we found that OST is sufficient to antagonize microglial activation in either LPS-induced BV2 cells or Aβ-overexpressing fruit flies. Again, silencing NRF2 abolishes OST’s antagonism, which means that NRF2 is necessary for this process. Furthermore, we found that OST treatment rescued survival, climbing, and the learning ability of AD fruit flies and relieved oxidative stress.

As described in the introduction, there are five other candidate pathways besides the NRF2 cascade for OST to antagonize microglial activation [17,18,19,20,21]. Could we exclude these candidates according to the results of this study? In Figure 3A, four microglial markers responded to OST treatment, while NRF2 RNAi abolished only three of them (Figure 3B). This may indicate that although the NRF2 cascade is the major pathway for OST to antagonize microglial activation, other minor contributing pathways are there. Thus, we cannot exclude the other five candidate pathways, but the NRF2 cascade is probably the key point.

In AD research, OST exhibits multiple benefits besides antagonizing microglial activation, including (1) decreasing Aβ cytotoxicity on neural cells by mediating phosphorylation of cAMP response element-binding protein (CREB) [30], (2) inhibiting apoptosis by mediating Wnt/β-catenin signaling [31], (3) improving synaptic plasticity and cognitive function by regulating the glutamatergic neuron [32], and (4) increasing hippocampal neurogenesis by upregulating Bdnf in AD mice [33]. Therefore, we could not exclude these factors and jump to the conclusion that OST improves AD by antagonizing microglial activation via the NRF2 cascade or by decreasing oxidative stress.

The KEAP1-NRF2 system is an evolutionarily conserved mechanism that controls not only the transcription of anti-oxidant, anti-inflammatory, and detoxifying proteins but also organism development [1]. For example, the KEAP1-knockout mice exhibit growth retardation, skin abnormalities, and early death after birth [34], and Cnc, the NRF2 homolog in Drosophila, controls development and head segment formation [35]. Therefore, the KEAP1-NRF2 system maintains a balance between excessive ROS scavenging and ROS-dependent signal transduction. In this sense, the therapeutic modulation of KEAP1-NRF2, such as OST treatment, must be carefully administered to attenuate inflammation-induced damage while leaving ROS-dependent signal transduction undisturbed. This may be a future challenge for the clinical application of OST to neurodegenerative diseases.

In conclusion, we proved that OST antagonizes microglial activation induced by either LPS or Aβ and that NRF2 is necessary for OST’s antagonism.

## 4. Materials and Methods

### 4.1. Osthole (OST)

The *Cnidium monnieri* source was identical to [36], and the purification method is described in [37], with a purity >98% as determined by HPLC, as described before [38]. OST forms a crystal of a light yellow color at room temperature. The stock solution was prepared at a concentration of 6 mg/mL in EtOH before use. For lifespan, climbing, learning ability, and gene expression assays, the final concentration of OST in the fruit fly medium was 6 μg/mL. For the BV2 cell culture, the OST concentration was 40 μM.

### 4.2. Western Blots

The western blotting assay detected specific proteins described previously [39]. In brief, cells were treated with OST 1 h before LPS. The proteins were extracted through PRO-PREP™ and separated by 8–12% SDS-PAGE. The proteins were transferred from the gel to the polyvinyl vinylidene fluoride (PVDF) membranes (Millipore Co., Billerica, MA, USA) and blocked with 5% BSA. Then, they were sampled with primary antibodies overnight and incubated with horseradish peroxidase (HRP) secondary conjugated antibody. The antibody detection response was performed using ECL. The antibody images were captured with the ImageQuant™ Las 4000 Mini Biomolecular Imager (GE Healthcare Life Sciences, Pittsburgh, PA, USA). The antibodies used were anti-Nrf2 (ab62352, Abcam), anti-phospho-Nrf2 (Ser40) (PA5-67520, Thermo Fisher, Waltham, MA, USA), anti-HO-1 (#5853, Cell Signaling, Danvers, MA, USA), anti-SOD1 (#2770, Cell Signaling), anti-Catalase (#14097, Cell Signaling), anti-IL1β (MA5-23691, Thermo Fisher), and anti-TNFα (ab183218, Thermo Fisher).

### 4.3. Immunofluorescence (IF) Assay

An IF assay has been used to visualize antibody specificity with fluorescent dyes in cells. Therefore, it makes it possible to visualize the distribution of the target protein through the sample of a fluorescence microscope, as described previously [40]. In brief, cells were incubated in a confocal laser dish (500 cells/dish) for 16 h and treated with OST before being incubated with 100 ng/mL of LPS. The cells were fixed in 4% paraformaldehyde and permeated with Triton X 100 at 0.25% in phosphate-buffered physiological serum (PBS). They were incubated for 1 h in 5% of PBS-BSA to block nonspecific binding. Moreover, cells were incubated with primary antibodies overnight at 4 °C and then complemented with a secondary antibody labeled with an IgG Alexa Fluor 488 and Alexa Fluor 594 reagent. They subsequently contaminated the nuclei with DAPI gel (1 μg/mL) in 1% BSA for 20 min at 37 °C in the dark. The IF staining images were visualized using a confocal SP2/SP8X spectral microscope (Leica Microsystems, Wetzlar, Germany).

### 4.4. Nrf2 siRNA Transfection

A Nrf2 siRNA transfection assay was used to investigate the antioxidant activity Nrf2, as described above [40]. In short, BV2 microglial cells were cultured in 6-well plaques (2105 cells/wells). Transfecting the DNA fragment encoding Nrf2 siRNA or Nrf2-negative siRNA control was carried out using LipofectamineTM 3000 (Invitrogen, Waltham, MA, USA). Mouse siRNA Oligo Duplex was used for the transfection of small interference RNAs (siRNAs). Nrf2 siRNA to knock down endogenous Nrf2 following the manufacturer protocol (Invitrogen). After 24 h, the transfected cells were exposed to OST (40 μM) and LPS for 24 h, followed by western blot and further analysis.

### 4.5. SOD/Catalase Activity

The SOD activity was detected with a Superoxide Dismutase Assay Kit (706002, Cayman), and the Catalase activity was detected with a Catalase Assay Kit (707002, Cayman), following the manufacture’s manual. The total protein was extracted from 50 flies for one replicate, and four replicates for OST treatment and nontreatment were collected, respectively. Protein was quantified with bovine serum albumin (BSA, New England BioLabs, Ipswich, MA, USA). The input quantity for SOD and Catalase activity was 0.125 μg and 10 μg, and the absorbance was measured at 450 nm and 540 nm, respectively.

### 4.6. Lipid Hydroperoxide (LPO) Assay

For one replicate, 100 flies were collected for the measurement of the total lipid hydroperoxide using BIOXYTECHÒ LPO-586 (OxisResearch), following the manufacture’s manual, except that the absorbance was measured at 595 nm. Three replicates were assayed.

### 4.7. Quantitative PCR

For Drpr and CED detection, the total RNA was extracted from 100 heads of flies for one replicate with an RNeasy Mini kit (Qiagen, Hilden, Germany), as previously described [41], and then used for synthesizing cDNA with a VersoTM cDNA Synthesis Kit (Thermo Scientific, Waltham, MA, USA). RNA expression levels of the investigated genes were quantified by real-time PCR (Applied Biosystems 7700) with the Maxima SYBR Green qPCR Master Mix (Thermo Scientific). The qPCR readouts were normalized to the relative amount of gapdh. Four independent measurements were performed.

For expression profiling of SOD/catalase genes, the total RNA was extracted from 50 flies for one replicate with PureZOL (Bio-Rad, Hercules, CA, USA), following the manufacturer’s manual. The reagents for reverse transcription and qPCR were identical to the previous section. The qPCR readouts were normalized to the relative amount of Rpl32.

For expression profiling of BV2 cells, the total RNA was extracted from cell lysates with PureZOL (Bio-Rad), following the manufacturer’s manual. The qPCR readouts were normalized to the relative amount of Actin. The primers used are listed in Supplementary Table S1.

### 4.8. Fly Stock Maintenance, Lifespan and Antigeotaxis Assays

The first familial offspring of the cross of the pan-neural Gal4 driver elav-GAL4^c155^ strain (Bloomington Drosophila Stock Center ID: 458) with UAS-Aβ42H29.3 [28] was utilized as the Alzheimer’s disease model. Only male flies were used. The flies were maintained in cornmeal standard media at 25 °C under a 12 h light-dark cycle. In a lifespan analysis, about twenty flies of the AD model were raised in a food vial with 6 μg/mL of OST, and four vials were prepared for each treatment. Food vials were replaced every 2 to 3 days, and dead flies were counted at that time. The percentage of survival is defined as the number of flies alive divided by the total number of flies at the beginning of the test. In the antigeotaxis assay to measure the climbing activity of *Drosophila*, flies were transferred into a new food vial just before measurement. The number of flies climbing more than 5 cm above the bottom in 18 s after being bounced to the bottom of vertically standing vials was counted. The percentage of climbing ability is defined as the number of climbing flies divided by the total number of flies at the beginning of the test.

### 4.9. Drosophila Learning and Memory Platform (T-Maze)

To observe *Drosophilae’s* learning and memory, about 100 flies were first placed in the upper cupper chamber, which can be supplied with electric voltage and deliver an electric shock to flies. Flies were first delivered with one odor, 4-methylcyclohexanol (MCH, 98%, Sigma Aldrich), along with electric shock waves at 80 V (Grass S88 stimulator, A-M Systems). Then, flies were delivered with another odor, 3-octanol (OCT, 97%, Sigma Aldrich), without electric shock waves. After training, the flies were moved to the space below, whose two open sides were supplied with either odor one or odor two, respectively. By observing the moving direction of flies, the bias of odor two over odor one can be estimated with the Performance Index (PI), which is defined as the difference of the number of flies between two odors divided by the total number of flies. The PI represents Drosophilae’s ability for learning and memory [42]. In this study, the time interval between the training session and the test session was 45 sec. The number of replicates of the learning assay was 14 and 6 for AD without treatment and for AD with 6 μg/mL of OST treatment for 14 days, respectively.

### 4.10. Statistical Analysis

The significance of the difference between the survival curves and climbing curves of OST-treated and control groups was judged by the log-rank (Mantel–Cox) test and Gehan–Breslow–Wilcoxon test. The significance of the difference between the PI of learning or memory of OST-treated and control groups was judged by Student’s *t*-test. Differences between LPS-treated and control groups were considered statistically significant at a level where the *p*-value was below 0.05. Statistical significance used the two-part Student *t*-test or One-Way Variance Analysis (ANOVA), as described previously [43], to determine statistical significance using the SPSS17.0 software system (IBM, Chicago, IL, USA). Differences between LPS-treated and control groups were considered statistically significant at a level where the *p*-value was below 0.05.

## Data Availability

The data presented in this study are openly available in FigShare at https://figshare.com/articles/figure/OST_NRF2_molecules/21780383 (accessed on 26 December 2022).

## Supplementary Materials

The following supporting information can be downloaded at: https://www.mdpi.com/article/10.3390/molecules28020507/s1, Table S1: Primers used in this study.

## Sample Availability

Samples of the compound osthole is available from the authors.
