# Supplementary material for: Osthole Antagonizes Microglial Activation in an NRF2-Dependent Manner
Source: Molecules. 2023 Jan 4;28(2):507. doi: 10.3390/molecules28020507 (PMC9912252; doi:10.3390/molecules28020507)
Supplement: Supplementary file 1 [file molecules-28-00507-s001.zip › Supplementary Table S1.pdf]

Osthole Antagonizes Microglial Activation in an NRF2-Dependent Manner

Chuan-Hsiu Liu [1,2,†], Mei-Ying Chen [3,†], Yueh-Hsiung Kuo [4,5,6], Jack Cheng [3,7], Li-Zhong Chang [3],

Meng-Shiun Chang [3], Tsai-Ni Chuang [3], Wen-Tsong Hsieh [4,8], Yan-Ru Xiao [8], Bor-Tsang Wu [9], Wei-Yong Lin [3,7,*] and Hsin-Ping Liu [10,*]

1 Graduate Institute of Chinese Medicine, China Medical University, Taichung 40402, Taiwan

2 School of Chinese Medicine, China Medical University, Taichung 40402, Taiwan

3 Graduate Institute of Integrated Medicine, College of Chinese Medicine, China Medical University, Taichung 40402, Taiwan

4 Department of Chinese Pharmaceutical Sciences and Chinese Medicine Resources, College of Chinese Medicine, China Medical University, Taichung 40402, Taiwan

5 Department of Biotechnology, Asia University, Taichung 41354, Taiwan

6 Chinese Medicine Research Center, China Medical University, Taichung 40402, Taiwan

7 Department of Medical Research, China Medical University Hospital, Taichung 40447, Taiwan

8 School of Medicine and Department of Pharmacology, China Medical University, Taichung 40402, Taiwan

9 Department of Senior Service Management, National Taichung University of Science and Technology, Taichung City, 40343

10 Graduate Institute of Acupuncture Science, College of Chinese Medicine, China Medical University, Taichung 40402, Taiwan

* Correspondence: linwy@mail.cmu.edu.tw (W.-Y.L.); hpliu@mail.cmu.edu.tw (H.-P.L.); Tel.: +886-4-22053366#3310 (W.-Y.L.)

† These authors contributed equally to this work.

Supplementary Table S1. The primers used in this study.

Organism	Gene	Forward primer	Reverse primer
Fruit fly	Gapdh	GAAAAAGCGGCAGTCGTAAT	AATTCCGATCTTCGACATGG
	Drpr	TGTGATCATGGTTACGGAGGAC	CAGCCGGGTGGGCAA
	CED	CGTTTACAAGGAGCGACT	TTCCCAGATTGAAGAGCAGG
	Rpl32	CGGATCGATATGCTAAGCTGT	CGACGCACTCTGTTGTCG
	Sod1	CCTCACCGGAGACCTTCAC	CAAGGGCACGGTTTTCTTC
	Sod2	AATTTCGCAAACTGCAAGC	TGATGCAGCTCCATGATCTC
	Sod3	TCAGCATGGGTGCTCACTAT	TAATGCCCGTGGAGTTGG
	Cat	TGACTACAAAAACTCCCAAACG	TTGATTCCAATGGGTGCTC
	CG15116	ACCGTTCGAGATACGTTTGG	CGCAAACCGTTGTACTGAGA
	PHGPx	TGACATCGGCGAGGTGT	CGGTCTGCTTGGCCTTTA
	Cnc	GCCAACTATGGTGGTGGAGT	ACGCTGCGATTCAGACG
Mouse	Actin	CATGAAGATCCTGACCGAGCGTG	TCTGCTGGAAGGTGGACAGTGAGG
	Nrf2	TTCTTTCAGCAGCATCCTCTCCAC	ACAGCCTTCAATAGTCCCGTCCAG
	IL1b	CTTCCTTGTGCAAGTGTCTG	CAGGTCATTCTCATCACTGTC
	Tnfα	AAATTCGAGTGACAAGCCTGTAG	GAGAACCTGGGAGTAGACAAGGT
	Ccl2	GGGATCATCTTGCTGGTGAA	AGGTCCCTGTCATGCTTCTG
	Ccl3	GTGGAATCTTCCGGCTGTAG	ACCATGACACTCTGCAACCA
	Cxcl1	CTTGACCCTGAAGCTCCCTT	AGGTGCCATCAGAGCAGTCT
	Ccl4	GCTCTGTGCAAACCTAACCC	GAAACAGCAGGAAGTGGGAG
	Ccl12	TCCTCAGGTATTGGCTGGAC	CGGACGTGAATCTTCTGCTT
